# Influence of sonication, temperature, and agitation, on the physical properties of a palm-based fat crystallized in a continuous system

**DOI:** 10.1016/j.ultsonch.2021.105550

**Published:** 2021-04-07

**Authors:** Thais Lomonaco Teodoro da Silva, Sabine Danthine, Silvana Martini

**Affiliations:** a Department of Nutrition, Dietetics, and Food Sciences, Utah State University, 8700 Old Main Hill, Logan, UT 84322-8700, USA; b Science des Aliments et Formulation, Gembloux Agro-Bio Tech, ULiège, Gembloux, Belgium

**Keywords:** High-intensity ultrasound, Agitation, Temperature, Sonocrystallization, Scraped surface heat exchanger

## Abstract

High-intensity ultrasound (HIU) has been used in the past to change fat crystallization and physical properties of fat crystalline networks. The objective of this work was to evaluate how HIU placed on different positions in a scraped surface heat exchanger (SSHE) using different processing conditions affect the physical properties of an interesterified palm olein. The sample was crystallized at two temperatures (20 °C and 25 °C) and two agitation rates (344/208 rpm and 185/71 rpm, barrels/pin worker). HIU (12.7 mm-diameter tip, 50% amplitude, 5 s pulses) was placed at three different positions within the SSHE. After processing, samples were stored at 25 °C for 48 h and analyzed according to the crystal morphology, solid fat content (SFC), oil binding capacity (OBC), melting behavior, viscoelasticity, and hardness. Physical properties were affected by crystallization conditions, by sonication, and by HIU position. The greatest improvement obtained was at 20 °C using low agitation when HIU was placed at the beginning of the SSHE. These conditions result in a sample with 98.9% of OBC, 274 kPa of viscoelasticity and 31 N of hardness. These results show that HIU can be used as an additional processing tool to improve physical properties of a palm-based fat and that the best improvement was obtained as a combination of crystallization conditions and HIU position.

## Highlights

• Position where HIU should be applied is dependent on temperature and agitation.
• At lower crystallization temperature, sample must be sonicated at HIU-1.
• At higher crystallization temperature, sample must be sonicated at HIU-3.
• HIU was able to improve fat physical properties such as G’ and OBC.
• Better physical properties were obtained 20 °C under low agitation in position HIU-1.

## 1 Introduction

Palm oil production is one of the largest worldwide, with>75 million tons produced in 2019 [1]. Palm oil has a unique fatty acid and triacylglycerol (TAGs) composition with approximately 50% saturated fatty acids and 50% unsaturated fatty acids which makes it suitable for numerous food applications [2]. Due to its unique composition various modification processes such as blending, hydrogenation, fractionation, and interesterification allow palm oil to be used in many edible and non-edible products [3]. The crystallization of palm oil and palm oil products has been extensively investigated to control the final quality of food products [4], [5], [6], [7], [8].

Crystallization happens in two steps: nucleation and crystal growth. To induce crystallization the molten fat is cooled to a temperature below the melting point to generate appropriate supercooling and induce the nucleation of high melting point TAGs. After nucleation, crystals grow and the amount of crystalline material increases [9]. Many internal and external factors affect the crystallization process. Internal factors include fat composition and presence of impurities while external factors include crystallization temperature, supercooling, agitation rate, use of ultrasound waves and others [10]. Comparing ultrasound with mechanical agitation, ultrasound can provide more uniform mixing and can avoid undesirable zones of excessive super saturation in the vessel [11]. Above and beyond, ultrasound can be used to induce nucleation where spontaneous primary nucleation cannot occur [12]. The cavitation and induced crystallization in the metastable zone will vary in different ways depending on many parameters such as the nature of the ultrasound source and its location; also, the influence of the cavitation is a function of the particular medium to which this form of energy is applied [13]. Previous studies have shown that the effect of sonication is affected by whether the acoustic waves are applied in the presence or in the absence of crystals, close or far from the onset of crystallization, with and without agitation [14], [15], [16], [17]. Previous studies have also shown that HIU efficiency changes depending on the fat composition [12], [18], [19], [20], [21]. This is particularly important when trying to incorporate HIU in a scraped surface heat exchanger (SSHE) since the position at which HIU is applied and other processing conditions such as agitation and crystallization temperature might affect ultrasound efficiency. Previous studies from our group have evaluated the use of HIU in a SSHE for a soybean-based fat and palm-based fat with low levels of saturated fatty acids using different sonication conditions and constant SSHE conditions. The HIU was coupled to the SSHE at different steps and using different sonication conditions. These studies showed that placing HIU at the end of the SSHE with a 50% amplitude pulses improved the physical properties of the fat [22], [23], [24], [25]. However, as previously mentioned SSHE conditions in the previous studies were maintained constant and it was unknown if and how changing SSHE parameters would affect HIU efficiency. That is, there is a need to explore if parameters in the SSHE can be changed to affect crystallization together with sonication ones. The combination of these factors in a commercially available palm-based sample has not been explored yet. Thus, the objective of this work was to evaluate how HIU placed on different positions in a SSHE using different crystallization processing conditions affect the physical properties of a palm-based fat.

## 2 Material and methods

### 2.1 Material

Interesterified palm olein (IEPO) was donated by ADM. The specification sheet reported a melting point (MP) between 38 and 44 °C. Fatty acid composition of 41.7% oleic, 39.6% palmitic acid, and 10.8% of linoleic acid and 4.3% stearic acid. Solid fat content values at different temperatures were 51.6 ± 0.01% at 10 °C, 29.2 ± 0.04% at 20 °C, 13.8 ± 0.01 at 30 °C and 5.19 ± 0.2% 40.0 °C, according to direct AOCS method Cd 16b-93 [26].

### 2.2 TAG composition

Reversed-phase HPLC based on the official AOCS method Ce 5b-89 [26] was performed to investigated the TAG composition. A Waters HPLC system (Zellik, Belgium), equipped with two stainless steel Nova-Pak C18 columns (4 lm, 3.9 × 150 mm) (Waters, Belgium) was used, with some minor adjustments to the flow rate and mobile phase composition. The mobile phase was a mixture of acetone and acetonitrile (62.5:37.5). The flow rate of 1.2 mL/min with 20 µl injection volume was used. For sample preparation, 0.04 g of sample was dissolved in 1.6 mL of methanol/chloroform (1:1 v:v) and the HPLC operated with a differential refractometer detection system. Peak areas were correlated with the quantities of TAGs in the oil or fat sample, and below 4,000 area counts (equivalent to approximately 0.04% of the total peak area) the peaks were not considered. The program Empower Pro with a generic Apex Track method was used for integration.

### 2.3 Crystallization

**Fig. 1:**
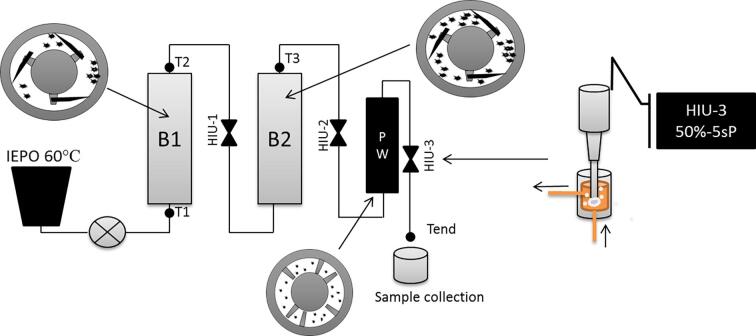
Schematic of the scraped surface heat exchanger crystallization process and high-intensity ultrasound positions (HIU-1, HIU-2, and HIU-3) in the system. B1: barrel one, B2: barrel two, PW: pin worker, T1, T2, T3, and Tend were temperature control points.

IEPO was completely melted in a stainless-steel pot placed on top on an induction oven set at 60 °C. The pot was connected to a scraped surface heat exchanger (SSHE, Armfield Limited, England, model FT25BBPA-IF-C) composed of a progressive cavity pump with a 20 L/h capacity (01-71L/4 TF), two barrels and a pin worker [25]. The melted sample was pumped into the system at 11*L*/h resulting in a residence time of 8 min. The capacity of the SSHE used in this study is 1.5L. The effect of crystallization temperature was evaluated by setting the wall temperature in the barrels to 20 or 25 °C. These temperatures were chosen based on previous study on small pilot scale SSHE [22], and on preliminary results performed in our laboratory to obtain SFC values at the end of the line higher than 5% for the highest temperature tested, to assure that nucleation took place inside of the SSHE, as designed in some industrial SSHE pilot plants. The effect of agitation was studied by setting the agitation in the barrels/pin worker at either higher agitation (344/208 rpm, HA) or low agitation (171/85 rpm, LA), agitation was chosen based on previous work [24]. High-intensity ultrasound (HIU) 20 kHz Q500 system (Qsonica, Newtown, CT, USA) was connected to a 65 mL water jacket flow cell (flow cell #4650; Qsonica, Newtown, CT, USA) and placed at different positions within the SSHE (HIU-1, HIU-2 and HIU-3) (Fig. 1). In HIU-1 the sonicator was placed between the two barrels; in HIU-2 the sonicator was placed after the second barrel, and in HIU-3 the sonicator was placed after the pin worker. HIU was applied using a 12.7 mm-diameter tip, 50% amplitude (57 W) and 5 s pulses (5 s ON/5 s OFF) while the sample was being pumped through the system.

Three temperatures were measured in the product during the process to monitor the cooling efficiency (Fig. 1). The first temperature measured was the one before barrel 1 (T1, 43.9 ± 1.3 °C), this temperature was similar to all conditions tested since it was measured before any cooling or sonication effect. The second thermocouple was placed after barrel 1 (T2) and indicates the cooling efficiency of this barrel. T2 slightly changed based on the crystallization temperature and agitation used but not due to HIU since this thermocouple was placed before HIU-1 (20HA = 21.7 ± 0.1 °C; 20LA = 23.1 ± 0.2 °C; 25HA = 27.0 ± 0.1 °C, 25LA = 27.4 ± 0.3 °C). The last temperature measured was labeled T3, and corresponds to a thermocouple located after barrel-2. This thermocouple is the point where sample achieves exactly the temperature set up and only showed a difference due to temperature used (T3, 20.0 ± 0.0 °C or 25.0 ± 0.0 °C). Each processing condition was performed three times and about 100 g of sample was collected and stored at 25 °C for 48 h (except for texture analysis = 5 °C for 48 h) before further analysis. Physical properties of the samples were measured after storage. Non-sonicated samples collected while processing the samples in the SSHE were used as a controls.

### 2.4 Methods

#### 2.4.1 Polarized light microscopy

After crystallization in the SSHE and storage at 25 °C for 48 h, one drop of the sample was placed on a slide and covered with a cover-slide. The slides were placed on a polarized light microscope – PLM (Olympus BX41 microscope, Olympus, Tokyo, Japan) with a digital camera attached to it (Infinity 2, Lumenera Scientific, Ottawa, ON, Canada). Images taken were used to characterize crystal morphology and to calculate the diameter of the crystals, crystallized area, and the number of crystals in a single image using the software Image-Pro® Premier E 9.2 (Media Cybernetics, USA). Crystal diameter was obtained as the mean of the measurements of the longer diameter of the different measured crystals. The crystallized area was estimated as the percentage of bright crystals in each image; the number of crystals is the mean of the counted crystals for each image of each crystallization condition. Four images were obtained for each crystallization run; the mean of the diameters was calculated using 12 different images.

#### 2.4.2 Oil binding capacity

One gram of the crystallized sample stored for 48 h at 25 °C was placed in a pre-weighed microcentrifuge tube and was centrifuged at 10,000 rpm for 15 min at room temperature using a microcentrifuge (Thermoscientific, Sorvall micro 17 centrifuge, Germany). After centrifugation the microcentrifuge tube was inverted upside down and left to drain for 3 min to release the liquid oil released from the crystalline network [27]. The oil binding capacity (OBC) was measured in quadruplicate for each crystallization run and calculated according to equation (1).

(1) $$OBC=100-(\frac{\left(Wbefore-Wafter\right)}{\left(Wsample\right)}*100)$$

where, *W_before_* (g) is the weight of the tube + sample before centrifugation, *W_after_* (g) is the weight of the tube + sample after centrifugation and drainage of the liquid oil, and *W_sample_ (g)* is the amount of sample placed in the tube before centrifugation.

#### 2.4.3 Melting behavior

The melting profile and melting point were measured in a Tzero DSC pan (10–15 mg of sample), covered with a Tzero lid and sealed hermetically. DSC was calibrated at 5 °C/min using Indium as a standard. To measure the sample’s melting point DSC parameters were based on the AOCS official method Cj 1–94 [26]. The pan containing the sample was placed in the DSC and kept at 25 °C for 1 min for stabilization, and then melted at 5 °C/min to 80 °C, kept at 80 °C for 30 min to erase crystalline memory, and cooled to −20 °C at 5 °C/min for complete crystallization. Samples were held at −20 °C for 90 min and melted to 80 °C at 5 °C/min. The peak temperature (T_p_) obtained from the last melting step was used to quantify the melting point [28].

To evaluate the melting behavior of the samples processed in the SSHE, stored samples (25 °C, 48 h) were stabilized at 25 °C for 1 min in the DSC and melted from 25 to 80 °C at 5 °C/min. The parameters used to quantify the melting behavior of the samples were onset temperature (T_on_), peak temperature (T_p_), and change in enthalpy associated with the melting process (ΔH). All DSC measurements were performed in triplicate.

#### 2.4.4 Viscoelasticity

The viscoelasticity of the sample crystallized in the SSHE and stored at 25 °C for 48 h was measured using an AR-G2 Rheometer (TA Instruments, New Castle, Delaware) operating with air purge (30 psi). Measurements were performed using a 40 mm standard steel parallel plate and a 5,000 μm gap. The instrument was operated by the Rheology Advantage Instrument Control software, where viscoelastic properties such as elastic (G′) and viscous (G″) moduli were measured using a strain sweep oscillation from 0.0008 to 10% strain at 25 °C. The frequency was kept constant at 1 Hz and G’ reported are means of the values obtained in the linear viscoelastic region (LVR). The Rheology Advantage software (TA Instruments, New Castle, Delaware) was used for data analysis. All experiments were performed in quadruplicate for each run.

#### 2.4.5 Texture profile analyzer

Hardness was measured for samples stored at 5 °C for 48 h in quadruplicate for each crystallization run. The samples were stored in 1 cm diameter plastic tubes. After storage, samples were removed from the plastic tubes and cut into 1 cm height cylinders. These cylinders were used to measure the hardness using a 2-step 25% compression test, using a 5 cm cylinder probe. The hardness of the samples was measured with a Texture Profile Analyzer (Model TA, XT Plus, Texture Technologies Corp., Hamilton, MA, United States).

#### 2.4.6 Solid fat content (SFC)

Samples were collected in nuclear magnetic resonance (NMR) tubes right after crystallization in the SSHE and after storage at 25 °C for 48 h. SFC was measured using a time-domain NMR (120 Minispec NMR analyzer, Bruker, Rheinstetten, Germany). Three tubes of each crystallization run were collected. The AOCS direct method Cd 16b-93 was used [26].

#### 2.4.7 Statistical analysis

Crystallization runs were performed in triplicates and analytical measurements were performed in replicates as described above. Data reported are mean values and standard deviations. Statistical differences between treatments were evaluated using 2-way ANOVA for crystallization temperature and HIU position (data on Table 2) and 3-way ANOVA for all physical properties measured. The factors evaluated in the 3-way ANOVA were agitation, crystallization temperature and HIU position. Tukey (α = 0.05) was used as a post-hoc test (GraphPad Prism, version 8.0, La Jolla, CA) for all analyses. Correlation analysis was also performed using GraphPad Prism version 8.0 (La Jolla, CA).

## 3 Results and discussion

### 3.1 Chemical composition and meting point

The TAG chemical composition is shown in Table 1. The IEPO in this study is mainly composed by POO and POP, 23.71 ± 0.41% and 23.48 ± 0.52%, respectively (P: palmitic acid, O: oleic acid), followed by POL (12.66 ± 0.10%, L: linoleic acid), and by other TAG components in concentrations between 8 and 5% such as PPP, PLP, OOL, OOO and POSt (St: stearic acid). The majority of TAGs were monosaturated (SUU, 40.07 ± 0.07%, S: saturated fatty acids, U: unsaturated fatty acids), followed by disaturated (SUS, 37.03 ± 0.39%), triunsaturated TAGs (UUU, 14.34 ± 0.08%) and trisaturated TAGs (SSS, 9.59 ± 0.20%).

**Table 1:** Triacylglycerol (TAG) composition of interesterified palm olein.

TAGs	Mean (%)
LLL	0.16 ± 0.22
LLO	1.03 ± 0.04
PLL	1.23 ± 0.06
OOL	5.64 ± 0.09
POL	12.66 ± 0.10
PLP	6.89 ± 0.32
MPP	0.48 ± 0.09
OOO	7.52 ± 0.11
POO	23.71 ± 0.41
POP	23.48 ± 0.52
PPP	7.09 ± 0.08
StOO	2.47 ± 0.13
POSt	4.70 ± 0.22
PPSt	2.01 ± 0.19
StOSt	0.71 ± 0.75
Total of TAGs by group	
UUU	14.34 ± 0.08
SUU	40.07 ± 0.32
SUS	37.03 ± 0.39
SSS	9.59 ± 0.20

P: Palmitic acid, St: stearic acid, O: Oleic Acid, L: Linoleic acid, Ln: Linolenic acid, M: Myristic Acid. S: Saturated fatty acids, U: Unsaturated fatty acids

**Table 2:** Crystallization and sonication parameters measured during and right after crystallization in the scraped surface heat exchanger (SSHE).

HIU position	B speed (rpm)	PW speed (rpm)	Pressure (bars)	Power level (W)	T_col_ (°C)	SFC (%)
				20 °C HA		
wo HIU-1	344	208	1.21 ± 0.06 ^bc^	0.00 ± 0.00 ^e^	21.33 ± 0.35 ^i^	19.97 ± 0.49 ^ab^
with HIU-1				101.33 ± 4.04 ^a^	21.77 ± 0.12 ^hi^	19.26 ± 0.52 ^ab^
wo HIU-2			1.38 ± 0.02^b^	0.00 ± 0.00 ^e^	21.70 ± 0.17 ^ghi^	20.10 ± 0.32 ^a^
with HIU-2				82.33 ± 0.58^b^	24.80 ± 0.20 ^cd^	18.03 ± 1.02 ^bcd^
wo HIU-3			2.55 ± 0.13 ^a^	0.00 ± 0.00 ^e^	21.40 ± 0.20 ^i^	19.67 ± 0.38 ^ab^
with HIU-3				70.67 ± 1.16^c^	23.63 ± 0.35 ^ef^	18.83 ± 1.10 ^abc^

	20 °C LA					
wo HIU-1	185	71	1.25 ± 0.07 ^bc^	0.00 ± 0.00 ^e^	21.13 ± 0.23 ^i^	17.29 ± 0.89 ^cde^
with HIU-1				104.00 ± 3.00 ^a^	19.80 ± 0.46 ^j^	16.60 ± 0.57 ^de^
wo HIU-2			1.10 ± 0.07 ^bc^	0.00 ± 0.00 ^e^	21.47 ± 0.51 ^i^	17.37 ± 1.31 ^cde^
with HIU-2				78.33 ± 1.16^b^	23.53 ± 0.45 ^efg^	14.35 ± 0.48^f^
wo HIU-3			2.31 ± 0.58 ^a^	0.00 ± 0.00 ^e^	20.73 ± 0.50 ^ij^	18.06 ± 1.08 ^bcd^
with HIU-3				69.67 ± 2.52^c^	22.67 ± 0.71 ^fgh^	15.56 ± 0.60 ^ef^

	25 °C HA					
wo HIU-1	344	208	0.61 ± 0.12 ^cd^	0.00 ± 0.00 ^e^	25.00 ± 0.00 ^cd^	8.93 ± 1.46 ^ghi^
with HIU-1				64.00 ± 0.00 ^d^	25.07 ± 0.06 ^cd^	7.92 ± 1.29 ^hij^
wo HIU-2			0.57 ± 0.09 ^cd^	0.00 ± 0.00 ^e^	25.83 ± 0.29^c^	10.39 ± 0.97 ^g^
with HIU-2				64.67 ± 0.56 ^d^	27.80 ± 0.27 ^a^	6.21 ± 0.80 ^jk^
wo HIU-3			1.10 ± 0.31 ^bc^	0.00 ± 0.00 ^e^	25.23 ± 0.06 ^cd^	9.23 ± 1.08 ^gh^
with HIU-3				62.67 ± 0.16 ^d^	27.87 ± 0.23 ^a^	7.34 ± 1.44 ^ijk^

	25 °C LA					
wo HIU-1	185	71	0.36 ± 0.06 ^d^	0.00 ± 0.00 ^e^	24.23 ± 0.40 ^de^	6.02 ± 0.15 ^jk^
with HIU-1				63.00 ± 2.65 ^d^	24.60 ± 0.44 ^de^	5.56 ± 0.29 ^kl^
wo HIU-2			0.43 ± 0.18 ^d^	0.00 ± 0.00 ^e^	24.53 ± 0.51 ^de^	6.10 ± 0.52 ^jk^
with HIU-2				62.00 ± 0.00 ^d^	26.53 ± 0.45^b^	3.00 ± 0.39^m^
wo HIU-3			0.68 ± 0.22 ^cd^	0.00 ± 0.00 ^e^	24.57 ± 0.31 ^de^	6.18 ± 0.25 ^jk^
with HIU-3				62.00 ± 0.00 ^d^	25.77 ± 0.40^c^	3.72 ± 1.18 ^lm^

*means followed by the same letter for the same parameter are not different from each other (α = 0.05). HIU: high-intensity ultrasound, B: barrels, PW: pin worker; HA: high agitation, LA: low agitation, wo: without HIU, with: with HIU, HIU-1: HIU position between two barrels, HIU-2: HIU position between barrel 2 and pin worker, HIU-3: HIU position at the end of the scraped surface heat exchanger (SSHE), Tcol: Temperature during sample collecting in the SSHE.

**Fig. 2:**
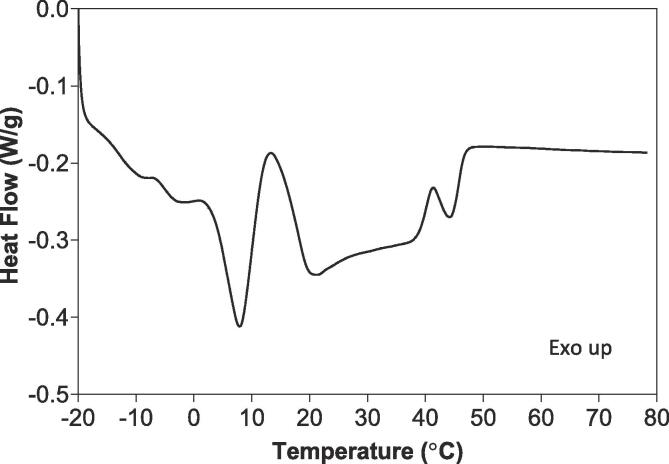
Melting curve of the interesterified palm olein (IEPO).

The melting profile obtained from the melting point measurement is shown in Fig. 2; the melting point calculated using the highest peak temperature (T_p_) was 44.6 ± 0.2 °C. This melting curve can be correlated to the chemical composition. The melting curve was formed by two main peaks that represent the heterogeneous TAG composition of this sample. The first peak is linked to the melting of SUU TAGs (~40%) that melt around 8 °C while the second peak corresponds to the melting of SUS and SSS TAGs. The second peak was subdivided in two shoulders. The first shoulder had a T_p_ around 21 °C and was linked to the melting of the SUS and the shoulder observed at 44 °C represents the melting of SSS TAGs [29].

### 3.2 Parameters of crystallization

During crystallization and sonication different parameters were monitored to evaluate their influence on the various physical properties (Table 2). Agitation rate and cooling temperature were the two variables changed in the SSHE. For clarity, HA was used to denote high agitation conditions of 344 rpm in both barrels and 208 rpm in pin worker (PW); and LA was used to denote low agitation corresponding to 185 rpm in the barrels and 71 rpm in the PW.

The pressure inside of the SSHE was not affected by sonication within the same temperature and agitation condition when HIU was applied in position HIU-1 and HIU-2. Higher pressures were obtained at 20 °C for both agitation rates when HIU was at HIU-3 compared to the pressure obtained at 25 °C. This difference in pressure occurs because the pressure is affected by the amount of crystalline material inside the SSHE, and more crystalline material was present at 20 °C compared to 25 °C. The pressure obtained in all other positions at 20 °C for HA or LA were similar to each other and similar to the pressure obtained at 25 °C HA HIU-1. The lowest pressure was obtained at position 1 and 2 using 25 °C HA and LA in all positions; these positions were the ones where less crystalline material was formed. This indicates that pressure changes due to changes in the HIU position is more related to the amount of crystalline material that must go through the flow cell than to the presence of the ultrasound cell.

The power level needed to achieve the same amplitude of tip vibration in the sonicator was affected by HIU position and crystallization temperature. For all temperatures and agitation conditions HIU-1 was the position were HIU needed more power. At HIU-1 power level was higher than 100 W, followed by HIU-2 with power levels of approximately 80 W and HIU-3 with power levels of approximately 70 W for both agitation rates. At 25 °C the power needed to reach the amplitude of vibration of the tip was lower (p < 0.05) than at 20 °C, and at 25 °C no differences in the power level between the different positions were found. In a previous study performed at higher temperatures (32 °C) the power was even lower (57 W) and no differences were found between the positions as reported above for 25 °C [24]. Higher power levels observed at lower temperatures are expected due to higher viscosity and more crystalline material formed. Higher cavitation thresholds are usually observed in samples with high viscosity [30].

The temperature of the samples as it came out of the SSHE (T_col,_ Table 2) changed according to crystallization temperature, but also changed due to agitation and HIU in some positions. At 20 °C HA, samples with HIU and without HIU in position HIU-1 were similar showing T_col_ around 21 °C (p > 0.05), when HIU was applied at positions HIU-2 and HIU-3, an increase in T_col_ was observed and this increase was higher at HIU-2 than at HIU-3 (p < 0.05). The increase in T_col_ at HIU-2 and HIU-3 could be due to (a) heat generated by cavitation events induced by sonication, (b) an induction in crystallization and the presence of heat of crystallization, or (c) combination of both events. Our hypothesis is that the increase in temperature is due mainly to cavitation events since the increase in temperature is associated with a slight decrease in SFC (Table 2). The greater increase in temperature observed for HIU-2 could be due to the higher power level observed in this condition compared to the HIU-3, since the higher increase in temperature usually occurs due to cavitation at higher power levels [31]. It is possible that a localized increase in temperature was also generated in HIU-1 position due to the high-power level observed in this condition, but this increase in temperature was dissipated through the SSHE and not detected at the end of the SSHE when the sample was collected.

At 20 °C LA, T_col_ of all non-sonicated samples were similar to each other and to all non-sonicated samples crystallized at 20 °C HA (p > 0.05). When HIU was applied at HIU-1 T_col_ was significantly lower than the one obtained in the non-sonicated samples (p < 0.05). This occurred because the SSHE is set up to maintain the sample temperature at the end of barrel 2 at 20 °C. When HIU was applied in this position the sample was slightly heated and the cooling system in the SSHE increases the cooling to maintain the temperature at 20 °C. This could have decreased the T_col_. This effect was more pronounced under LA conditions because in this condition the cooling is slower than in HA condition. Similar trends to the ones observed for 20 °C HA were observed for sonicated samples at HIU-2 and HIU-3.

At 25 °C both agitation rates showed similar tendencies, all non-sonicated samples and sonicated samples at position HIU-1 were similar (p > 0.05) with temperatures around 25 °C, and at positions HIU-2 and HIU-3 T_col_ increased due to sonication (p < 0.05). Previous studies did not find any difference in the T_col_ as found in this study [24], [25]. We believe this happened because in previous studies a different fat even with lower content of saturated fatty acids was crystallized at a higher temperature (32 °C). And although sample left barrel 2 at 32 °C, by the time it was collected the temperate went down to 25 °C only by going through the PW, even though the PW was not double walled. This means that due to the crystallization conditions used in these previous studies, the PW worker acted as an additional cooling step, not been possible to observe heating due sonication.

The last parameter evaluated during crystallization was SFC, which was affected by crystallization temperature, agitation, and sonication. In a previous study, agitation was the main factor responsible for the amount of solid fat (SFC) formed in the SSHE, followed by temperature [32]. Similarly, in our results the highest values for SFC (~20%) were obtained at 20 °C HA, which were all similar to each other independently of HIU position or HIU treatment (p > 0.05). The only exception found was for the sonicated sample at position HIU-2 which had a lower SFC and was the sample that showed the biggest increase in T_col_ suggesting that the increase in the temperature in this position due to cavitation partially melted the crystalline material. For 20 °C LA, the SFC was similar for all non-sonicated samples and for sonicated at position HIU-1 (~17%). A significantly reduction (p < 0.05) in SFC was found for sonicated samples at HIU-2 and HIU-3 (~14–15%) and the reduction was higher for HIU-2 which it was statistically similar to position HIU-3. When crystallizing at 25 °C, HA also promoted higher SFC than LA, and for both agitation conditions the trends were similar to those observed for 20 °C LA, where only sonicated samples at HIU-2 and HIU-3 showed smaller amount of solids and they were similar to each other. The SFC was correlated to the T_col_ (p > 0.001; r = -0.80).

### 3.3 Microstructure

**Fig. 3:**
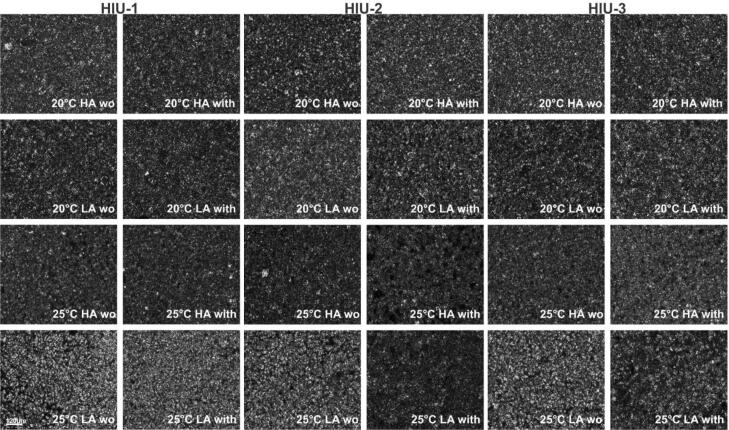
Polarized light microscopy images obtained after storage at 25 °C for 48 h for a palm-based sample crystallized under different conditions. For samples nomenclature please refer to Table 2.

The microstructure of the samples obtained under the different conditions is presented in Fig. 3. The characteristic parameters (crystal mean diameter, crystallized area and number of crystals) obtained from those images are shown in Table 3. Visually no differences were found in the crystal size and morphology using HA or LA at 20 °C. All crystalline networks were characterized by very small needle-like crystals that were attached to each other forming a well-connected crystalline network with very small amount of liquid material (dark spots) independently of HIU application and HIU position. Observing the data presented in Table 3, even though the crystals look the same, slightly bigger crystals were obtained at LA with HIU in position 2 and 3 where we also observed a higher T_col_. However, all samples using LA at 20 °C showed similar crystal diameter between them (p > 0.05).

**Table 3:** Crystal diameter (D), crystallized area (CA), and the number of the crystals counted after storage at 25 °C for 48 h.

HIU position		20 °C					
	HA			LA			
	D (µm)	CA (%)	Number of crystals	Dm (µm)		CA (%)	Number of crystals
wo HIU-1	4.78 ± 0.12 ^fgh^	10.16 ± 1.36 ^defg^	1100 ± 82 ^bcde^	4.80 ± 0.05 ^fgh^		10.01 ± 1.00 ^defg^	1134 ± 70^b^
with HIU-1	4.66 ± 0.07 ^h^	9.71 ± 0.81 ^efg^	1135 ± 68^b^	4.78 ± 0.09 ^fgh^		10.68 ± 1.10 ^defg^	1157 ± 60^b^
wo HIU-2	4.79 ± 0.05 ^fgh^	9.07 ± 0.67 ^g^	990 ± 51 ^cdef^	4.95 ± 0.13 ^ef^		11.56 ± 1.36 ^cde^	1175 ± 72^b^
with HIU-2	4.66 ± 0.08 ^h^	10.20 ± 0.60 ^defg^	1199 ± 51^b^	4.94 ± 0.11 ^ef^		11.66 ± 1.25 ^cde^	1146 ± 41^b^
wo HIU-3	4.80 ± 0.07 ^fgh^	11.40 ± 0.48 ^cdef^	1178 ± 74^b^	4.82 ± 0.07 ^fgh^		9.88 ± 0.75 ^efg^	1108 ± 95 ^bc^
with HIU-3	4.68 ± 0.06 ^gh^	11.02 ± 1.26 ^defg^	1196 ± 130^b^	4.96 ± 0.08 ^ef^		12.10 ± 0.94 ^cd^	1198 ± 53^b^

HIU position		25 °C					
	HA			LA			
	D (µm)	CA (%)	Number of crystals	Dm (µm)		CA (%)	Number of crystals
wo HIU-1	4.85 ± 0.06 ^fgh^	11.11 ± 0.91 ^defg^	1162 ± 66^b^	5.66 ± 0.27 ^a^		17.86 ± 2.01 ^a^	1352 ± 49 ^a^
with HIU-1	4.86 ± 0.10 ^fgh^	10.54 ± 0.57 ^defg^	1105 ± 24 ^bcd^	5.40 ± 0.27^b^		17.26 ± 2.19 ^ab^	1422 ± 53 ^a^
wo HIU-2	4.78 ± 0.06 ^fgh^	9.18 ± 0.77 ^g^	976 ± 45 ^defg^	5.30 ± 0.28 ^bc^		15.52 ± 1.12^b^	1210 ± 98^b^
with HIU-2	5.20 ± 0.15 ^bcd^	10.78 ± 1.33 ^defg^	926 ± 38 ^fg^	4.95 ± 0.11 ^ef^		9.04 ± 1.14 ^g^	971 ± 80 ^efg^
wo HIU-3	4.91 ± 0.03 ^efg^	10.95 ± 1.34 ^defg^	1144 ± 107^b^	5.37 ± 0.07^b^		13.27 ± 1.02^c^	1111 ± 52 ^bc^
with HIU-3	5.00 ± 0.07 ^def^	11.39 ± 1.01 ^cdef^	1090 ± 178 ^bcdef^	5.10 ± 0.08 ^cde^		9.49 ± 1.06 ^fg^	854 ± 76 ^g^

*means followed by the same letter for the same parameter are not different from each other (α = 0.05). For nomenclature please refer to Table 2.

The samples that presented bigger crystals also showed a higher amount of crystallized area as samples HIU-2 LA without HIU, and HIU-3 LA with HIU both at 20 °C. The increase in the crystal size in samples with LA happened because the SSHE has a scraping action that removes the crystals formed in the cooled surface and mixes them producing an abundance of small crystals, which accelerates further crystallization [33], [34]. The use of a higher agitation rate intensifies this process breaking down crystals and limiting crystal growth [35].

Crystal morphology was not affected by crystallization temperature as shown in Fig. 3. A tendency of fewer crystals obtained under HA conditions compared to the ones obtained under LA at 25 °C was observed. This visual tendency can also be observed in Table 3. At 25 °C using HA, crystals size was similar to 20 °C HA except for sonicated samples in HIU-2 (5.20 µm) and HIU-3 (5.00 µm) that showed bigger crystals compared to 20 °C LA too, but only the value obtained for HIU-2 was significantly higher (p < 0.05). Fewer differences were found between samples crystallized using 20 °C LA and HA and 25 °C HA. These samples had always a crystallized area between 9 and 12%. Crystals smaller than 4.8 µm showed crystallized area close to 9% and as crystal size increased to values close to 5 µm the crystallized area increased.

Samples crystallized at 25 °C under LA showed bigger crystals than all other crystallization conditions (p < 0.05) except for sonicated samples at positions HIU-2 and HIU-3 that showed smaller crystals, as the ones found using 20 °C LA (p > 0.05) at same positions and significantly smaller than samples crystallized at this crystallization condition (25 °C LA; p < 0.05). As observed in Fig. 3 samples sonicated in positions HIU-2 and HIU-3 also showed lower crystallized area (p < 0.05) than other conditions at the same T_c_ and agitation, and lower crystal sizes were attributed to smaller crystallized area. At position HIU-1 both non-sonicated and sonicated samples showed statically higher crystallized area than any other condition and HIU-2 without HIU was the only one that showed significantly higher area than the others (p < 0.05).

Crystal size and crystallized area (CA) were correlated to each other (p < 0.001; r = 0.82), suggesting that bigger crystals showed a bigger percentage of total crystallized area. CA was correlated with the number of crystals (p < 0.001; r = 0.76), more crystals contributed to a more crystallized area, but no correlation was found between the diameter and number of crystals (p > 0.05). Area crystallized was not correlated to any of the parameters measured during crystallization (p > 0.05). The diameter of crystals was correlated to SFC (p < 0.001, r = -0.71). The last correlation reinforces that lower SFC obtained immediately after the sample leaves the SSHE allowed crystals to grow during storage.

### 3.4 Solid fat content (SFC)

**Fig. 4:**
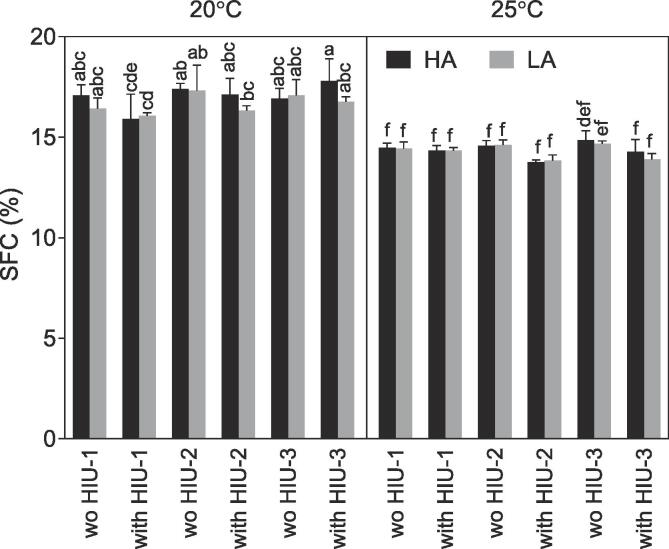
Solid fat content (SFC) obtained after storage at 25 °C for 48 h, for the palm-based sample crystallized under different conditions. For samples nomenclature please see Table 2.

SFC values obtained after storage at 25 °C for 48 h are presented in Fig. 4. SFC at 20 °C with HA or LA was always higher than the ones obtained at 25 °C (SFC ~ 14%, p < 0.05) and similar to each other (SFC ~ 17%, p > 0.05) except for samples sonicated at HIU-1 which were lower than HIU-2 without HIU and HIU-3 HA without HIU (p < 0.05). For samples crystallized at 25 °C, HIU position and agitation did not affect the SFC. Even though SFC was lower in sonicated samples during sample collection in positions HIU-2 and HIU-3 (Table 2), this difference was not observed after storage indicating that even though sonicated samples presented lower SFC at the beginning, they achieved the same final SFC over storage than non-sonicated. For example, the SFC of samples processed under HA conditions increased from ~ 8% to 14%, and at lower agitation (LA) this increase was even higher from < 6% to approximately 14% (Table 2, Fig. 4). Similar results were observed by Chen et al. [36] and Gregersen et al. [37] after storage of sonicated samples.

When samples were crystallized at 20 °C all solid material was already produced in the SSHE and no changes were observed due to 48 h storage. The exception was the sonicated samples at LA where the SFC increased by about 1–2% in positions HIU-2 and HIU-3 after storage. SFC after storage was correlated with SFC and temperature just after sample collection (p < 0.001 for both, SFC r = 0.94, T_col_ r = -0.77) and crystal size (p = 0.003, r = -0.59). These results indicate that higher SFC values obtained after storage were obtained when higher SFC and lower temperatures were observed in the SSHE process, and when small crystals were formed.

### 3.5 Oil binding capacity (OBC)

**Fig. 5:**
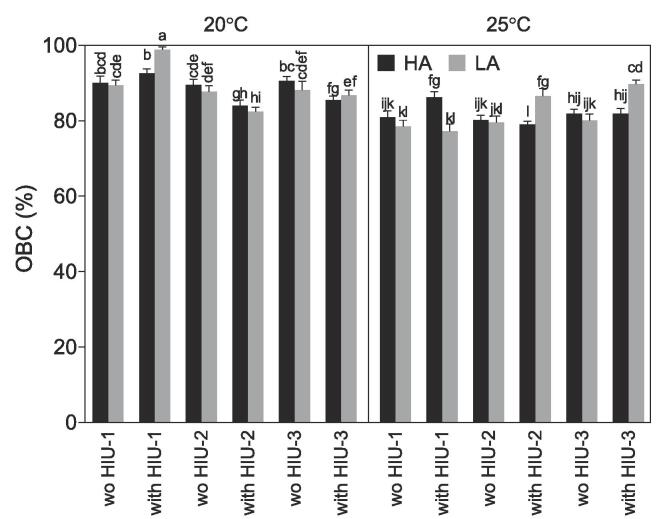
Oil binding capacity (OBC) obtained after storage at 25 °C for 48 h for the palm-based sample crystallized under different conditions. For samples nomenclature please refer to Table 2.

The OBC results are presented in Fig. 5. The highest OBC observed was for the sonicated sample at HIU-1 crystallized at 20 °C using LA (98.9 ± 0.6%), followed by the sonicated sample at same position crystallized at 20 °C using HA (92.6 ± 1.2%) that was significantly lower (p < 0.05). However, the OBC obtained using 20 °C HA is not significantly different from HA without HIU on positions HIU-1 and HIU-3 (~90%, p > 0.05). OBC in all non-sonicated samples were similar to each other when crystallized at 20 °C using the same agitation (p > 0.05). OBC in samples crystallized using LA was usually slightly lower than the ones obtained for samples crystallized using HA at 20 °C (p > 0.05). SSHE crystallization under a high agitation rate (1000 rpm) usually shows lower OBC than under lower shear (100 rpm) regardless of the temperature used due to the excessive stirring [28]. The similarity in the OBC in our study might be due to the lower shear used (maximum 344 rpm).

Sonication in other positions besides HIU-1 at 20 °C reduced the OBC of the samples instead of improving it. The reduction is visible for all positions but significant (p < 0.05) only for positions HIU-2 using HA or LA, and HIU-3 using HA, suggesting that the increase in temperature in this position negatively changed the crystalline structure formed resulting in a lower OBC.

Crystallization at 25 °C showed a lower OBC than at 20 °C that could be attributed to the lower amount of solid formed inside of the SSHE and to the partial crystallization during storage (Table 2). Nevertheless, HIU in this case never had a negative effect and either improved OBC values or did not change them compared to the non-sonicated samples which were always similar to each other (~80%, p > 0.05). When crystallized at 25 °C under HA, HIU improved the OBC in position HIU-1 (86.3 ± 1.4%) but this improvement was not as good as the one observed for HIU-1 at 20 °C for both agitation rates. When crystallized under LA conditions HIU improved OBC values at both positions HIU-2 (86.5 ± 2.0%) and HIU-3 (89.8 ± 1.1%). The best improvement obtained at 25 °C was found in position HIU-3 using LA (p < 0.05). Even though a significant improvement in OBC was observed in samples crystallized at 25 °C LA in position HIU-3, the OBC value obtained was similar to all controls obtained at 20 °C (p > 0.05). These results suggest that HIU can be used to match OBC values when samples are processed at high temperatures (25 °C) to OBC values obtained at low temperatures (20 °C). However, OBC can be improved even further when samples are processed at lower temperatures (20 °C) with the use of HIU (for example in position HIU-1).

It was assumed that the effect of sonication as a function of temperature and agitation rate is different because after cavitation bubbles are formed by the HIU the bubbles can either dissolve, collapse, coalesce, or expand and compress around their natural oscillation frequency over time [38]. When HIU is applied before the onset of crystallization (presence of few crystals), as in position HIU-1 for 25 °C LA, there are no nuclei formed and the bubbles were not able to induce primary nucleation and probably dissolved [24]. When HIU was applied at the onset of crystallization as is the case of HIU-1 for 20 °C LA and HA and 25 °C HA the cavitation bubbles generated were able to induce secondary nucleation resulting in a reduction in crystal size, better oil entrapment, and an improvement in physical properties [15], [16], [24], [25], [38]. When HIU was applied after the onset of crystallization, mainly for samples that experienced a higher supercooling (20 °C; positions HIU-2 and HIU-3) the localized increase in temperature generated by the collapsing bubbles might have melted the crystalline material in the SSHE forcing the fat to re-crystallize after it has been collected from the SSHE during storage at 25 °C without the scraping and agitation resulting in a lower OBC. This melting was confirmed by the data reported in Table 2 in the SFC and T_col_. Besides as observed in the OBC even after storage, sonicated samples in the wrong position never fully reached the desired OBC. In agreement with our theory the OBC was correlated with SFC collected and T_col_ (SFC p = 0.001, r = 0.62; T_col_ p < 0.001, r = -0.71) and was also correlated with crystal diameter, crystallized area and SFC after storage (p = 0.001, r = -0.63, p = 0.004, r = -0.57, p = 0.01, r = 0.52, respectively).

### 3.6 Viscoelasticity

**Fig. 6:**
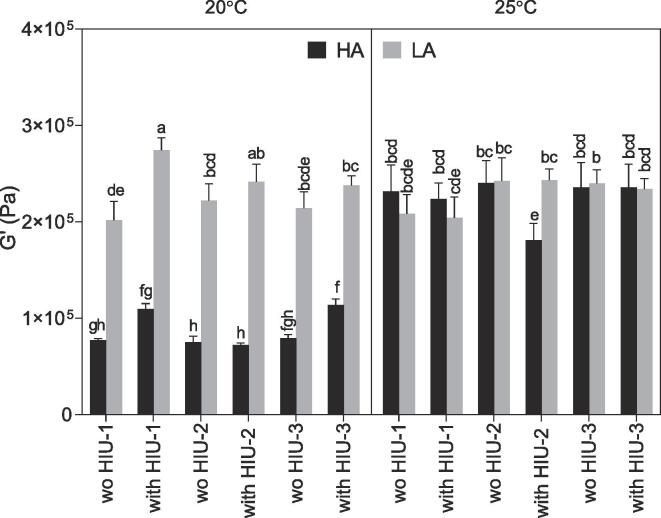
Elasticity (G’) obtained after storage at 25 °C for 48 h, for the palm-based samples crystallized under different conditions. For samples nomenclature please refer to Table 2.

Viscoelasticity is presented in Fig. 6. The most elastic (G’) sample was the one crystallized with sonication at 20 °C using LA at position HIU-1 (274 ± 12 kPa) which is the same condition that showed the best OBC. The G’ observed for sonicated samples at 20 °C LA HIU-2 was slightly lower but not significantly different (242 ± 18 kPa, p > 0.05). Almost all samples crystallized at 25 °C HA or LA and 20° LA showed similar G’ values and all controls were statistically similar (p > 0.05). Sonicated sample at 25 °C HA HIU-2 showed a reduced value compared to the other samples at 25 °C.

G’ values obtained for samples crystallized at 20° C HA were much lower compared to other samples (<100 kPa; p < 0.05), even though they were characterized by small crystals and good OBC. The use of a low shear rate (100 *vs* 1000 rpm) in the SSHE in a previous study has also shown higher viscoelasticity, even though the lower sheared samples showed lower SFC [32]. In our study, it is possible that the 20 °C LA samples were less crystallized compared to the HA samples (lower SFC in Table 2) and some additional crystallization occurred during storage that increased the G’ values. In addition, HA samples were subjected to higher agitation in the pin worker that might have also contributed to a less elastic sample. Similarly, Acevedo and Marangoni [39], found that agitation induced crystallization compared to a static crystallization, either using a scraped wall system or laminar agitation induced a significant decrease in the value of G’ without decreasing OBC, and the reduction in the G’ was more evident in the scraped system being attributed to the presence of blades that promotes a higher degree of crystal breakage. In this case, the authors even used a lower wall temperature than in this study (0 °C). Van Aken & Visser [35], also reported the negative effect of adding an additional agitation step to crystallize milk fat in a SSHE, where the continuous and excessive agitation instead of promoting crystal packing, avoids the crystals from bound to each other. It is possible that high shear, which also corresponds to high shear in the pin worker, breaks down molecular interactions formed during crystallization in the barrels resulting in lower elasticity. This behavior is not seen at 25 °C because the sample continues to crystallize during the pin worker and after storage (Table 2, Fig. 4). Interactions formed at this stage are the ones that drive elasticity values. G’ was correlated to SFC collected immediately after the sample left the SSHE (SFC_c_) or after storage (SFC_c_ p = 0.001, r = -0.63; SFC storage p = 0.003, r = -0.57) but was not correlated to OBC nor crystal size.

### 3.7 Hardness

**Fig. 7:**
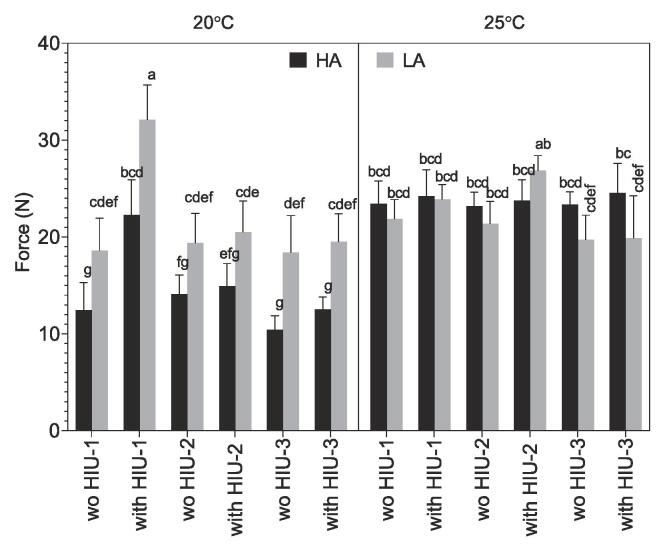
Hardness (N) obtained after storage at 25 °C for 48 h, for the palm-based sample crystallized under different conditions. For samples nomenclature please refer to Table 2.

Hardness is presented in Fig. 7 after storage at 48 h at 5 °C, and as observed for viscoelasticity the highest hardness was observed for samples sonicated in position HIU-1 using LA and 20 °C (32.1 ± 3.6 N). This high value of hardness was followed by the one obtained for samples crystallized at 25 °C LA with HIU at HIU-2, but this value was not different from other conditions tested at 25 °C.

Similar hardness was obtained for samples sonicated at 20 °C using LA conditions except for HIU-1 with HIU as discussed above. However, using HA at 20 °C, the hardness was quite lower as observed for viscoelasticity except for sonicated sample at position HIU-1. As discussed for G’ similar results were found due to the addition of extra agitation in milk fat at 20 °C, where the extra agitation reduced the firmness that did not recover even after storage [35]. Hardness was correlated to SFC collected (p = 0.003, r = -0.58) and after storage (p < 0.001, r = -0.67) and G’ (p < 0.001, r = 0.76).

### 3.8 Melting behavior

**Fig. 8:**
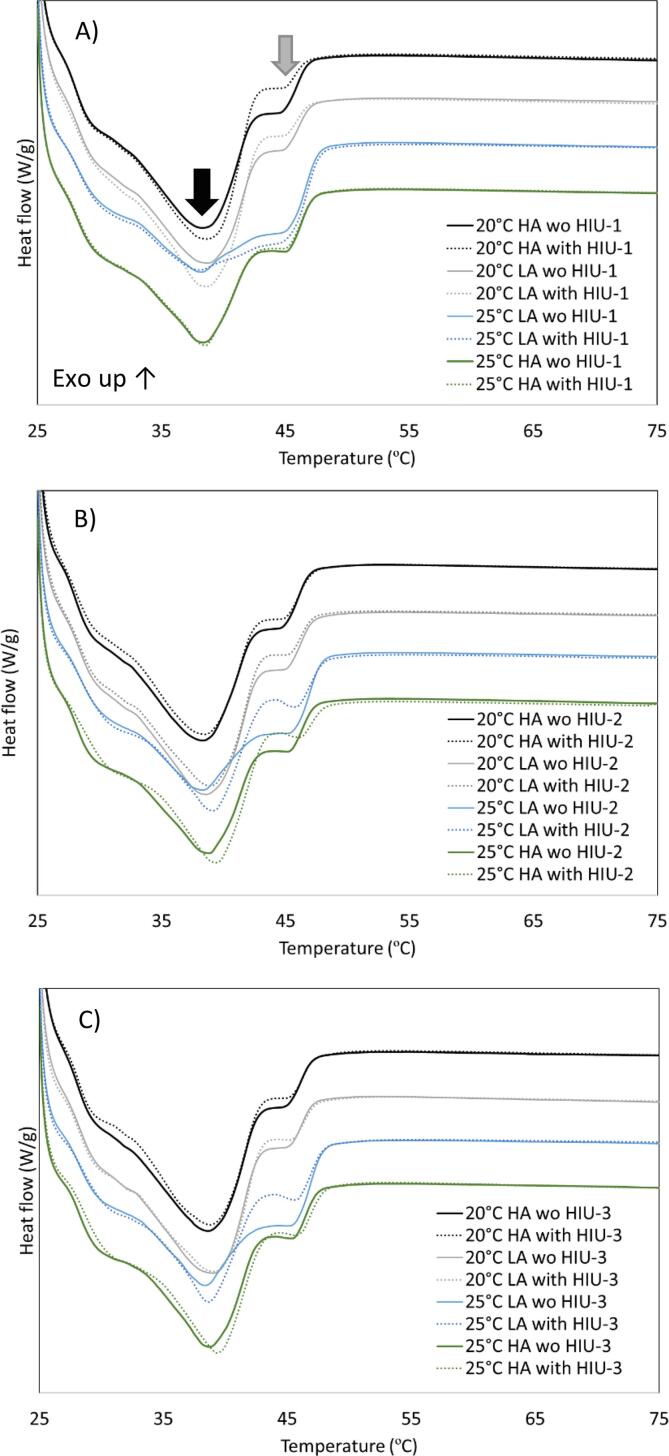
Melting behavior obtained after storage at 25 °C for 48 h, for the palm-based sample crystallized under different conditions. (A) samples at position HIU-1, (B) samples at position HIU-2 and (C) samples at position HIU-3. For samples nomenclature please refer to Table 2.

Fig. 8 and Table 4 show the results of the melting curves. Observing the shape of the curves (Fig. 8A), at position 1, all samples presented the same shape with a big peak that melts around 38 °C (black arrow) and a small well-defined shoulder with a peak around 45 °C (grey arrow), except for sample 25 °C LA with or without HIU, that showed a broader peak. Still, on position HIU-1, samples sonicated at 20 °C seem to show a smaller shoulder and bigger main peak than non-sonicated samples at the same position. Sample 25 °C HA does not show any change in the shape of the melting peaks due to sonication.

**Table 4:** Melting parameters obtained after storage at 25 °C using different crystallization conditions.

HIU position	20 °C					
	HA			LA		
	T_on_ (°C)	T_p_ (°C)	ΔH (J/g)	T_on_ (°C)	T_p_ (°C)	ΔH (J/g)
wo HIU-1	27.03 ± 0.07^b^	38.56 ± 0.34 ^cdef^	25.98 ± 0.89 ^abcd^	27.12 ± 0.35 ^ab^	38.87 ± 0.17 ^abcdef^	25.52 ± 1.17 ^abcde^
with HIU-1	27.06 ± 0.14^b^	38.84 ± 0.49 ^abcdef^	25.88 ± 1.03 ^abcde^	27.19 ± 0.13 ^ab^	38.74 ± 0.36 ^bcdef^	25.07 ± 0.92 ^bcde^
wo HIU-2	27.07 ± 0.18^b^	38.61 ± 0.37 ^cdef^	26.65 ± 1.03 ^ab^	27.25 ± 0.31 ^ab^	38.83 ± 0.35 ^abcdef^	26.48 ± 1.09 ^abc^
with HIU-2	27.48 ± 0.58 ^ab^	38.71 ± 0.26 ^cdef^	25.22 ± 1.65 ^abcde^	27.28 ± 0.45 ^ab^	39.09 ± 0.32 ^abcde^	24.70 ± 1.10 ^bcdef^
wo HIU-3	26.98 ± 0.21^b^	38.73 ± 0.51 ^bcdef^	27.42 ± 0.85 ^a^	27.32 ± 0.41 ^ab^	39.09 ± 0.31 ^abcde^	25.70 ± 1.03 ^abcd^
with HIU-3	27.28 ± 0.44 ^ab^	38.87 ± 0.54 ^abcdef^	25.79 ± 2.46 ^abcd^	27.05 ± 0.30^b^	39.27 ± 0.29 ^abc^	25.84 ± 0.86 ^abcd^

HIU position	25 °C					
	HA			LA		
	T_on_ (°C)	T_p_ (°C)	ΔH (J/g)	T_on_ (°C)	T_p_ (°C)	ΔH (J/g)
wo HIU-1	27.22 ± 0.13 ^ab^	38.54 ± 0.29 ^cdef^	22.76 ± 0.62 ^ef^	27.20 ± 0.03 ^ab^	38.37 ± 0.24 ^ef^	23.90 ± 0.67 ^cdef^
with HIU-1	27.20 ± 0.19 ^ab^	38.56 ± 0.19 ^cdef^	22.86 ± 1.00 ^ef^	27.20 ± 0.16 ^ab^	38.19 ± 0.38^f^	24.06 ± 0.94 ^bcdef^
wo HIU-2	27.19 ± 0.14 ^ab^	38.75 ± 0.19 ^abcdef^	23.00 ± 1.21 ^ef^	27.26 ± 0.26 ^ab^	38.34 ± 0.30^f^	24.64 ± 0.96 ^bcdef^
with HIU-2	27.22 ± 0.16 ^ab^	39.45 ± 0.25 ^a^	22.04 ± 0.94 ^ef^	27.18 ± 0.12 ^ab^	39.14 ± 0.20 ^abcd^	22.97 ± 1.05 ^ef^
wo HIU-3	27.15 ± 0.31 ^ab^	38.87 ± 0.26 ^abcdef^	23.90 ± 1.60 ^cdef^	27.17 ± 0.36 ^ab^	38.45 ± 0.25 ^def^	24.03 ± 1.11 ^bcdef^
with HIU-3	27.24 ± 0.29 ^ab^	39.42 ± 0.34 ^ab^	24.26 ± 2.10 ^bcdef^	28.61 ± 1.01 ^a^	38.82 ± 0.26 ^abcdef^	23.26 ± 1.53 ^def^

*means followed by the same letter for the same parameter are not different from each other (α = 0.05). For nomenclature please refer to Table 2.

Analyzing position HIU-2 (Fig. 8B), samples crystallized at 20 °C with and without HIU basically overlap. A very small reduction on the shoulder is observed on sample crystallized under HA with sonication and the same reduction on sample sonicated under LA as observed for position HIU-1 was detected, nevertheless, at position HIU-2 there was no change in the main peak. Samples crystallized at 25 °C showed a visible reduction in the shoulder size with sonication and a smaller increase in the main peak size. The changes observed for 25 °C HIU-2 samples due to sonication confirms that for 25 °C the induction of crystallization happened later than at 20 °C and HIU is more effective after crystallization occurs in both barrels as in the case of HIU-2. Last for samples crystallized at HIU-3, no changes in the peak profiles were observed except for 25 °C LA with HIU where the shoulder was reduced the most. The reduction in shoulder size and the increase in the main peak concur with the better OBC positions and viscoelasticity for each crystallization condition. This suggests that HIU in those positions might be inducing crystallization of lower melting points TAGs and/or forming a more homogeneous and compacted crystalline structure [12]. Cavitation generated by sonication induced bubbles formation, collapse, and shock waves promoted nucleation through decreasing critical energy for crystal formation and therefore inducing the crystallization of low melting point TAGs. In addition, acoustic-induced nucleation might permit better modulation of crystal growth [11].

Observing the melting parameters generally, all samples start melting at around 27 °C (T_on_, Table 4), have it maximum between 38 and 39 °C (T_p_, Table 4) and finish melting at around 47 °C (Fig. 8) and few or no statistical differences were found on these melting temperatures (p > 0.05). No changes on melting parameters due to HIU treatment in milk fat crystallized in a continuous system were previously found [37] nor in an IESBO crystallized in a SSHE [25], but changes in the melting behavior were found for palm oil where a reduction in T_p_ was observed in a sample crystallize without agitation [40].

Nevertheless, few changes were observed in the melting enthalpy (ΔH) due to crystallization conditions. In general, ΔH was lower for samples crystallized at 25 °C due to the lower amount of crystalline material formed. ΔH was correlated to many parameters such as SFC collected at the exit of the SSHE (p < 0.001, r = 0.87) and after storage (p < 0.001, r = 0.85), temperature during collection (p < 0.001, r = -0.80), OBC (p = 0.005, r = 0.55), G’ (p = 0.005, r = -0.56) and hardness (p = 0.001, r = -0.64).

## 4 Conclusion

Crystallization is an important step involved in the processing of fat-based products. Many parameters must be controlled such as temperature, agitation, and flow rate. HIU can be used as an additional processing tool to change fat crystallization. When product temperature is maintained at 20 °C under a low agitation rate (185 rpm/71 rpm) sonication affects significantly all physical properties of the fat such as OBC, viscoelasticity, and hardness when applied at the beginning of the crystallization (onset of crystallization). Cavitation was promoted to induction nucleation; enough nucleation was formed to seed the sample and withstood the crystallization process that occurred under shaved agitation.

The use of a higher agitation rate reduced the strength of the crystalline network, which might happen due to excessive agitation after the crystalline material was formed. At higher temperatures, the HIU position that worked the best was when applied later in the SSHE process due to the lower supercooling and a consequent delay in the onset of crystallization. The condition that resulted in better physical properties such as high OBC, G’, and hardness was when the samples were processed at 20 °C under LA in position HIU-1.

## CRediT authorship contribution statement

**Thais Lomonaco Teodoro da Silva:** Conceptualization, Data curation, Formal analysis, Investigation, Methodology, Writing - original draft, Writing - review & editing. **Sabine Danthine:** Data curation, Formal analysis, Resources, Supervision, Writing - review & editing. **Silvana Martini:** Conceptualization, Visualization, Funding acquisition, Methodology, Project administration, Resources, Supervision, Writing - review & editing.

## Declaration of Competing Interest

The authors declare that they have no known competing financial interests or personal relationships that could have appeared to influence the work reported in this paper.
